# Protocatechuic Acid Attenuates Inflammation in Macrophage-like Vascular Smooth Muscle Cells in ApoE^−/−^ Mice

**DOI:** 10.3390/nu17061090

**Published:** 2025-03-20

**Authors:** Shuangshuang Li, Yushi Du, Guanyu Chen, Yihui Mao, Wenyu Zhang, Mengxi Kang, Shasha Zhu, Dongliang Wang

**Affiliations:** 1 Department of Nutrition, School of Public Health, Sun Yat-sen University, Northern Campus, Guangzhou 510080, China; lishsh86@mail2.sysu.edu.cn (S.L.); duysh5@mail2.sysu.edu.cn (Y.D.); chengy268@mail2.sysu.edu.cn (G.C.); maoyh23@mail2.sysu.edu.cn (Y.M.); zhangwy69@mail2.sysu.edu.cn (W.Z.); kangmx@mail2.sysu.edu.cn (M.K.); zhushsh8@mail2.sysu.edu.cn (S.Z.); 2 Guangdong Provincial Key Laboratory for Food, Nutrition and Health, Guangzhou 510080, China; 3 Guangdong Engineering Technology Center of Nutrition Transformation, Sun Yat-sen University, Guangzhou 518107, China

**Keywords:** anti-inflammation, atherosclerosis, exportin-1, macrophage, nuclear factor kappa B, phenotypic plasticity, protocatechuic acid, vascular smooth muscle cells

## Abstract

**Background/Objectives**: Non-resolving inflammation in macrophage-like cells (MLCs) transdifferentiated from vascular smooth muscle cells and monocyte-derived macrophages aggravates atherosclerosis. We previously showed that polyphenolic protocatechuic acid (PCA) could reduce inflammation burden in monocyte-derived macrophages; however, it remains unknown how this compound affects MLCs inflammation. **Methods**: MLCs from the transdifferentiation of vascular smooth muscle cells induced by cholesterol and 30-week-old male ApoE^−/−^ mice fed a semi-purified AIN-93G diet containing either 0.003% (wt:wt) of PCA for a duration of 20 weeks were used to examine the impact of PCA on the inflammatory response of MLCs. **Results:** Physiologically achievable doses of PCA (0.25–1 μM) dose-dependently inhibited lipopolysaccharide-induced NF-κB activation and simultaneously reduced pro-inflammatory cytokine levels. Mechanistically, this effect was mediated by effecting exportin-1 function, promoting nuclear export of phosphorylated-p65, independent of NF-κB kinase inhibitor α/β/γ, NF-κB inhibitor α, or importin-mediated nuclear import of *p*-p65. PCA reduced the nucleocytoplasmic ratio of exportin-1 (44%) without altering its abundance. Importantly, dietary supplementation with PCA reduced interleukin-1β content within MLCs in atherosclerotic plaques of ApoE^−/−^ mice. In addition, dietary PCA reduced MLCs content in atherosclerotic plaques. **Conclusions**: PCA could attenuate inflammatory response in MLCs by targeting exportin-1 and also could inhibit the transdifferentiation of vascular smooth muscle cells into MLCs within atherosclerotic plaques, which might promote the translation from preclinical studies to clinical trials in patients with atherosclerosis.

## 1. Introduction

Atherosclerosis remains a major contributor to global cardiovascular morbidity and mortality [1]. The driving factor for atherosclerosis is a complex interaction between vascular cells, lipids, and inflammatory mediators [1]. One strategy that has been extensively studied but remains central to atherosclerosis reduction is identifying new ways to promote the resolution of inflammation within atherosclerotic plaques [2]. Apart from monocyte-derived macrophages, accumulating evidence has suggested that resolution of inflammation in vascular smooth muscle cells (VSMCs) inhibits atherosclerosis development [3].

Some of the pivotal cells in the pathology of atherosclerosis are VSMCs [3], which have traditionally been thought to play a role in maintaining vascular tone and forming the protective fibrous cap of atherosclerotic plaques [4,5]. However, recent clinical and preclinical studies have strongly suggested that VSMCs have a significant plasticity, including a transdifferentiation into macrophage-like cells (MLCs) with either a protective or deleterious effect in atherosclerotic plaque progression [6,7,8,9]. MLCs have been documented to contribute up to 50% of macrophages within atherosclerotic plaques, which is in contrast to the prevailing concept that macrophages in atherosclerotic plaques are predominantly of myeloid monocyte origin [10,11,12]. Functionally, MLCs partially resemble the functions of monocyte-derived macrophages, such as phagocytic capacity, foam cell formation, and lysosomal acid lipase activity [13]. They can also exhibit the proinflammatory phenotype in response to inflammatory stimuli [4,14]. Therefore, the improved function of MLCs within atherosclerosis plaques is a promising strategy to protect against atherosclerosis.

The nuclear factor kappa B (NF-κB), which mainly consists of p65 and p50, serves as a pivotal regulator of inflammatory cascades in MLCs. In addition to the well-recognized upstream regulators in the cytoplasm, such as the NF-κB kinase inhibitor (IKK) α/β/γ and NF-κB inhibitor α (IκBα), the transport efficiency of phosphorylated-p65 (*p*-p65) between the nucleus and cytoplasm is also essential in the activation of the NF-κB signaling pathway. On the one hand, the import of cytosolic *p*-p65 to the nucleus has been attributed predominantly to importin α1, α3, and α4 [15,16]. On the other hand, the export of nuclear *p*-p65 to the cytoplasm was mediated by exportin through the nuclear pore complexes. Exportin-1 and exportin-2, two members of the nuclear export machinery, are highly responsible for recognizing and exporting *p*-p65 [17,18].

Diet, a critical lifestyle factor, plays a fundamental role in the pathogenesis of atherosclerosis. Both clinical and preclinical studies have shown that dietary polyphenols, widely existing in plant foods, are protective factors for atherosclerosis [19,20]. For example, anthocyanidins and proanthocyanidins could reduce the risk or severity of atherosclerosis [21,22]. These atheroprotective effects are likely due to polyphenols themselves and also to these gut microbiota phenolic acids, including protocatechuic acid [23,24]. Mechanistically, studies involving in vitro cell cultures and studies on experimental animal models and subjects have strongly suggested that various biological activities, such as anti-oxidation, anti-inflammation, and enhancement of apoptotic cell clearance, contribute to these atheroprotective properties [25,26,27,28,29]. Among these mechanisms, the anti-inflammatory effects of polyphenols on macrophages within atherosclerotic plaques have garnered our significant attention.

Protocatechuic acid (3,4-dihydroxybenzoic acid), a naturally occurring phenolic acid, is widely distributed in plant foods, such as teas, fruits (plum and berry), vegetables (lettuce and French endive), seasoning (star anise and rosemary), vegetable oils (acai oil and extra virgin olive oil), and medicinal herbs (*Radix et Rhizoma Salviae Miltiorrhizae*, *Acanthopanax senticosus*, and *Eucommia ulmoides*). It is also a gut microbiota metabolite of some polyphenols, such as anthocyanins, quercetin, and proanthocyanidins [24,30]. Vitaglione Paola et al. showed that plasma concentrations of protocatechuic acid peaked at 492 nM within two hours after six healthy volunteers consumed blood orange juice containing 71 mg of anthocyanin glucoside [31]. Several other flavonoids have also been reported to be metabolized into protocatechuic acid [30]. Direct sources from plant foods and indirect sources from gut microbiota metabolism of flavonoids thus highlight the nutritional value of protocatechuic acid.

Protocatechuic acid has been documented to have a wide range of health-promoting effects, such as anti-oxidation, anti-inflammation, and anticancer [32,33,34]. Our previous work and others have found that protocatechuic acid could inhibit atherosclerosis development and reduce inflammation burden in whole macrophage populations within atherosclerotic plaques in apolipoprotein E-deficient (ApoE^−/−^) mice [24,35,36,37,38,39]. Consistently, previous studies confirmed the anti-inflammation property of protocatechuic acid and revealed regulatory pathways using monocyte-derived macrophages challenged with different stimuli that exist in atherosclerotic plaques [37,40,41]. However, no studies have yet evaluated the effect of protocatechuic acid on inflammatory response specifically in MLCs within atherosclerotic plaques. Investigating protocatechuic acid in MLCs may uncover novel pathways distinct from those in monocyte-derived macrophages, refining our understanding of the inflammation in atherosclerosis.

To this end, the primary objective of this study was to explore the effect of protocatechuic acid on the inflammatory response in MLCs, while the secondary objective was to explore whether protocatechuic acid affects the transdifferentiation of VSMCs into MLCs.

## 2. Materials and Methods

### 2.1. Isolation and Culture of Primary VSMCs

The isolation and culture of VSMCs were conducted as previously described [42]. Primary cells were harvested from the aortic tissue of 4-week-old C57BL/6J mice (Guangdong Province Medical Experimental Animal Center, Guangzhou, China). Following isolation, cells were maintained in DMEM medium supplemented with 10% fetal bovine serum and an antibiotic mix containing 100 units/mL penicillin and 100 µg/mL streptomycin (all from Gibco, Grand Island, NY, USA). To preserve phenotypic stability, only cells with fewer than five passages were utilized for subsequent experiments. The purity of VSMCs was over 98% as determined by immunocytochemistry with an α-actin antibody.

### 2.2. Transdifferentiation of VSMCs into MLCs

Based on previous studies [11,43], VSMCs were cholesterol-loaded with cholesterol and methyl-β-cyclodextrin complexes (Chol:MβCD; 10 µg/mL; Sigma-Aldrich, St. Louis, MO, USA) for 72 h to transdifferentiate into MLCs. To characterize the phenotypic changes, Western blot, qRT-PCR, and immunocytochemistry assays were used to detect VSMC markers (α-actin and smooth muscle 22α) and macrophage markers (F4/80 and ATP-binding cassette transporter A1).

### 2.3. Cell Treatments

To evaluate the effect of protocatechuic acid (purity (HPLC) ≥ 98%; Sigma-Aldrich) on the inflammation of MLCs and the transdifferentiation of VSMCs into MLCs, cells were treated with 0–1.0 µM of protocatechuic acid in the presence or absence of Chol:MβCD (10 µg/mL) for 72 h. According to the reference [44], 5 nM inhibitor leptomycin B (LMB, Cayman Chemicals, Ann Arbor, MI, USA) was used to inhibit exportin-1 for 4 h prior to stimulating with or without 50 pg/mL lipopolysaccharide (LPS) for 1 h (Sigma-Aldrich), which was explored to aggravate the inflammatory response.

### 2.4. Dosage Information

VSMCs and MLCs were exposed to escalating concentrations of protocatechuic acid (0.25–1.0 µM), the doses that can be achievable in blood circulation through a regular human diet (~100 g of Brussels chicory for a 60 kg person) [45]. Indeed, protocatechuic acid at 0.25 to 1.0 µM has been reported to significantly modulate the phenotype of several cell types related to atherosclerosis development, such as an increase in macrophage cholesterol efflux and efferocytosis [24,38], and a reduction in endothelial inflammation [46].

### 2.5. Mouse Study

All experimental protocols, including tissue samples, were adopted from our previous study conducted in accordance with the ARRIVE 2.0 guidelines (Table S1) [35,47]. In brief, 30-week-old ApoE^−/−^ male mice (57BL/6J background) that had been fed a semi-purified AIN-93G diet for 25 weeks were randomly allocated into two groups: a control group fed an AIN-93G diet (*n* = 15) and an experimental group maintained on the same diet containing 0.003% (wt:wt) of protocatechuic acid (*n* = 15). The number of animals is based on experience from studies of atherosclerosis in ApoE^−/−^ mice [24,37,38]. Fresh food was served every three days. Five mice per cage were housed in a specific pathogen-free room with a regular 12:12 h light/dark cycle (lights on at 07:00 a.m.), at a constant room temperature of 22 ± 2 °C, and relative humidity approximately 55 ± 10%. There were no exclusions. After 20 weeks, mice were fasted overnight and sacrificed after inhalational anesthesia of isoflurane. Hearts were collected and stored at −80 °C until use. The animals were analyzed in a random order to minimize the influence of confounding factors. Blinding was not implemented in this study. The animal protocol was approved by the Ethics Committee of the School of Public Health at Sun Yat-sen University [No. 2017–001].

To measure the inflammation burden in MLCs in atherosclerotic plaques, transverse sections (8 μm thickness) of the brachiocephalic artery were prepared and subjected to dual immunohistochemical staining using primary antibodies against F4/80, Caldesmon, and/or interleukin (IL)-1β. Identification of MLCs from the atherosclerotic plaque was guided by the double staining of F4/80 and Caldesmon (a calmodulin binding protein), which exhibited negligible expression in monocyte-derived macrophages within plaques, but robust expression was observed in VSMC independent of cholesterol loading, not like α-actin [11]. These slides were used as “guide slides” for the preceding and subsequent adjacent slices. In adjacent sections, MLCs were located at the same position as in the guide section with Caldesmon^+^ according to reported methods [48]. Nuclei were stained by DAPI. Confocal imaging was performed using a laser scanning confocal microscope (LSM 900; ZEISS). The antibodies employed in this study are detailed in Table 1. The Ethics Committee of the School of Public Health at Sun Yat-sen University has granted approval for all animal procedures [No. 2017-001].

### 2.6. Cell Viability Assay

Cell viability was assayed by 3-(4,5-dimethylthiazol-2-yl)-2,5-diphenyltetrazolium bromide (MTT) and lactate dehydrogenase (LDH) assay. VSMCs and MLCs were treated with protocatechuic acid for 24 h. The MTT assay, quantifying mitochondrial reductase activity, was conducted following established protocols [37]. Concurrently, membrane integrity was evaluated by measuring LDH release using an LDH Assay Kit (Cytotoxicity) (Abcam, Cambridge, UK), with supernatant collection and enzymatic quantification strictly adhering to the manufacturer’s standardized protocol.

### 2.7. RNA Preparation and Quantitative Real-Time PCR

Total RNA was isolated from cultured cells by using RNeasy Kits (Qiagen, Hilden, Germany). Subsequently, complementary DNA was synthesized via reverse transcription with a SuperScript III kit (Invitrogen, Waltham, MA, USA). qRT-PCR was then conducted to quantify mRNA levels using iQ™ SYBR^®^ Green Supermix (Bio-Rad, Hercules, CA, USA) on a QuantStudio™ 6 Pro Real-Time PCR System (Thermo Fisher Scientific, Waltham, MA, USA). The specific primer sequences used for each gene are provided in Table 2. Relative expressions were calculated by the comparative threshold cycle method and presented as a relative expression ratio 2^(−ΔΔCt)^. Glyceraldehyde-3-phosphate dehydrogenase (GAPDH) mRNA levels served as an internal control.

### 2.8. Protein Extraction and Western Blot

Protein extraction was performed to analyze whole-cell, cytoplasmic, and nuclear lysates. Whole-cell proteins were extracted using RIPA lysis buffer (Solarbio, Beijing, China), while nuclear and cytoplasmic fractions were isolated using a commercial extraction kit (Thermo Fisher Scientific) to ensure compartment-specific protein purity. Protein concentrations were determined through the BCA protein assay kit (Novagen, Pretoria, South Africa) before separating by SDS-PAGE. Following electrophoretic separation, proteins were electrotransferred onto PVDF membranes. The antibodies employed in this study are detailed in Table 1. Band intensities were quantified using the automatic digital gel imaging analysis system (Tanon-5200; Tanon, St Andrews, UK) and ImageJ 1.54 software (NIH). Normalization was performed against GAPDH (cytoplasmic loading control) or histone H3 (nuclear marker), depending on subcellular localization.

### 2.9. Inflammatory Cytokines Analysis

The levels of interleukin (IL)-1β, IL-6, and tumor necrosis factor (TNF)-α in the cultured supernatant from VSMCs and MLCs were measured using mouse IL-6 (M6000B; R&D Systems), IL-1β (MHSLB00; R&D Systems), and TNF-α (MTA00B; R&D Systems) ELISA kits, following the manufacturer’s protocols.

### 2.10. DNA-Binding Activity of Nuclear p65

To evaluate the DNA-binding capacity of p65 in the nucleus—a critical step in NF-κB transcriptional activation—nuclear extracts were isolated and prepared from cultured MLCs using the protocol detailed earlier. Subsequently, the DNA-binding activity of nuclear p65 was quantified using a TransAM NF-κB p65 ELISA kit (Active Motif, Carlsbad, CA, USA), following the manufacturer’s protocol.

### 2.11. Immunocytochemistry

VSMCs and MLCs were fixed at room temperature for 20 min using 4% formaldehyde and subsequently permeabilized in PBS containing 0.5% Triton X-100 (Sigma-Aldrich). Following a 1-h blocking step with normal donkey serum, the cells underwent treatment with mouse anti-α-actin (1:1000 dilution), rat anti-F4/80, or rabbit anti-*p*-p65 in blocking buffer overnight. This was followed by a 2-h incubation in the dark with fluorescent secondary antibodies (all from Invitrogen). Following secondary antibody incubation, cells were incubated with DAPI (Invitrogen) to counterstain the nuclei. To minimize observer bias, confocal imaging was performed under blinded conditions. Two independent, trained operators, unaware of experimental group assignments, acquired high-resolution images using a laser scanning confocal microscope (LSM 900; ZEISS, Jena, Germany).

### 2.12. Small Interfering RNA (siRNA)-Dependent Knockdown of Exportin-1

The cells were reverse-transfected with either a non-targeting scrambled siRNA control or *Exportin-1*-targeting siRNA (*si-Exportin-1*) (1027417, Qiagen) using Lipofectamine RNAiMAX Transfection Reagent (Invitrogen). The efficiency of the siRNA treatment was determined by both qRT-PCR and Western blot assays.

### 2.13. Statistical Methods

Data were expressed as mean ± SEM. Normality was tested by the Kolmogorov-Smirnov test. Differences between two groups were made by unpaired Student’s two-tailed *t*-test (animal experiments). Group comparisons were made by one-way ANOVA coupled with Tukey’s (variance homogeneity) or Games-Howell’s (variance heterogeneity) post hoc test for multiple comparisons (cell cultures) using SPSS 19.0 (IBM) and GraphPad Prism Version 9.4.0. Differences with *p* < 0.05 were considered significant.

## 3. Results

### 3.1. Protocatechuic Acid Attenuates Inflammatory Response in MLCs

**Figure 1:**
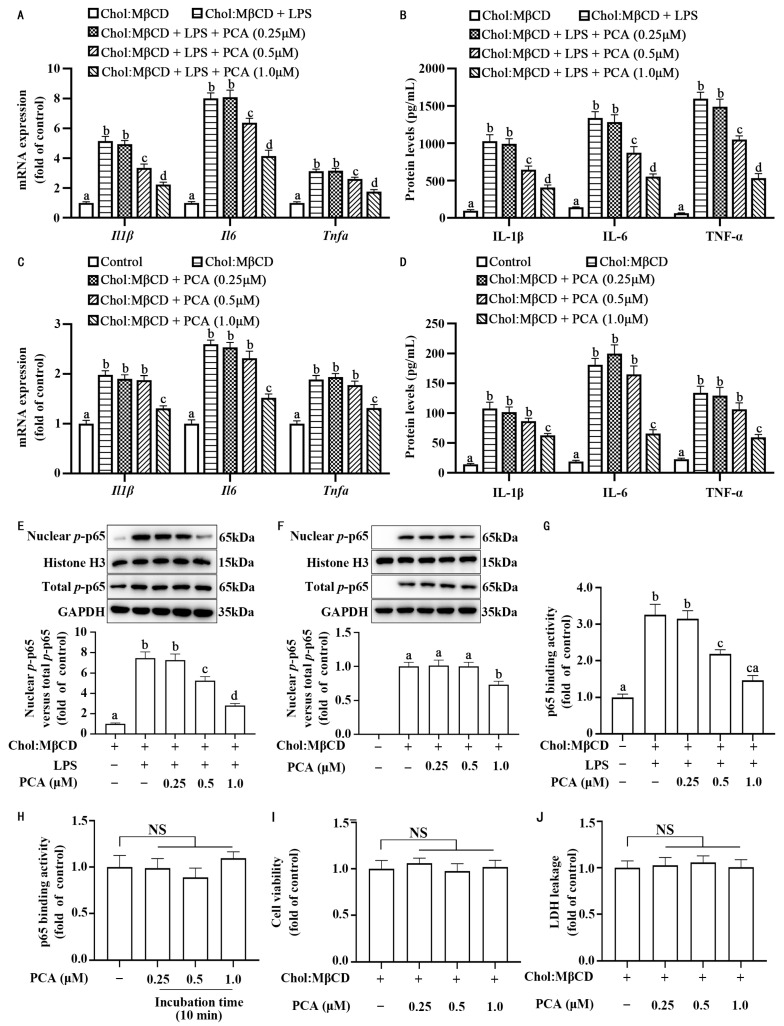
Protocatechuic acid attenuates inflammatory response in MLCs. (**A**,**C**) The mRNA expression of *Il1β*, *Il6*, and *Tnfα* in MLCs, and (**B**,**D**) IL-1β, IL-6, and TNF-α protein concentrations in the culture medium of MLCs incubated with protocatechuic acid for 72 h with or without LPS, respectively. (**E**,**F**) Representative Western blot of *p*-p65 protein expression and densitometric quantification of *p*-p65 normalized to GAPDH and Histone H3. (**G**,**H**) *p*-p65 binding activity with DNA. (**I**) MTT assay for MLCs viability. (**J**) Measurement of LDH leakage. Values are shown as mean ± SEM. *n* = 6. Meanings associated with labels lacking shared letters exhibited significant divergence (*p* < 0.05). NS, nonsignificant (*p* ≥ 0.05).

**Figure 2:**
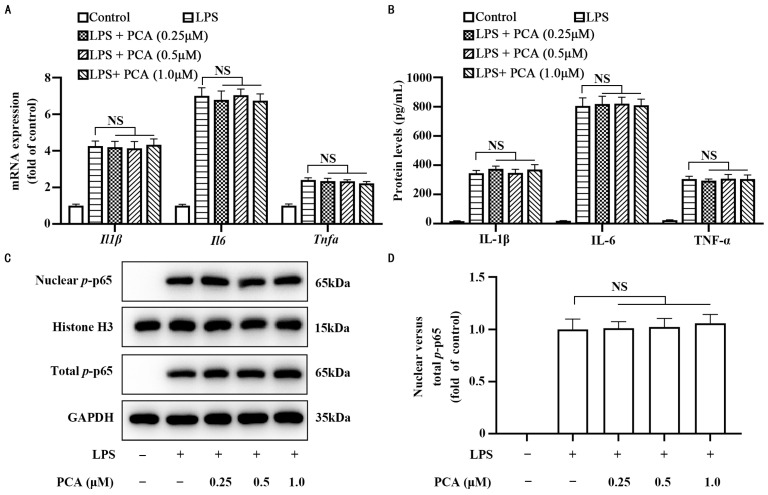
Protocatechuic acid does not modulate inflammatory response in VSMCs. (**A**) The mRNA expression of *Il1β*, *Il6*, and *Tnfα* in VSMCs incubated with protocatechuic acid for 72 h. (**B**) IL-1β, IL-6, and TNF-α concentrations in the culture medium of VSMCs. (**C**) Representative Western blot of nuclear and total *p*-p65 protein expression and (**D**) densitometric quantification of *p*-p65 normalized to GAPDH and Histone H3. Values are shown as mean ± SEM. *n* = 6. *p* < 0.05. NS, nonsignificant (*p* ≥ 0.05).

To assess the impact of protocatechuic acid on inflammatory response in MLCs, primary mouse aortic VSMCs were cholesterol-loaded with Chol:MβCD for 72 h to transdifferentiate these cells into MLCs. Protocatechuic acid dose-dependently inhibited the inflammation response, as evidenced by a reduction in IL-1β, IL-6, and TNF-α at the mRNA (Figure 1A,C) and protein levels (Figure 1B,D) in MLCs stimulated or unstimulated with 50 pg/mL of LPS, a reachable level in patients or laboratory animals with atherosclerosis [49,50]. Moreover, this compound also reduced the content of nuclear *p*-p65 (Figure 1E,F), which was associated with a reduction in the binding activity of nuclear *p*-p65 to the consensus NF-κB binding motif (Figure 1G). To test whether protocatechuic acid directly affects the DNA-binding activity of nuclear *p*-p65, we prepared nuclear extract from MLCs stimulated with LPS and divided the nuclear proteins equally into two parts. One section underwent treatment with protocatechuic acid for a duration of 10 min, whereas the other section received treatment with the solvent DMSO. As shown in Figure 1H, the binding activity exhibited no significant difference, suggesting that protocatechuic acid did not directly affect the binding ability of the p65 to DNA. In addition, the effect of protocatechuic acid on inflammatory response in MLCs was associated with no significant change in cell survival assessed by both MTT and LDH assays (Figure 1I,J). In order to verify whether the anti-inflammatory effect of protocatechuic acid depends on the activation state of VSMCs, we examined its effect on inflammatory response in naive VSMCs. As shown in Figure 2A,B, protocatechuic acid did not appreciably affect the mRNA and protein levels of IL-1β, IL-6, and TNF-α, as well as nuclear *p*-p65 content (Figure 2C,D). These findings thus suggest that protocatechuic acid selectively attenuates inflammatory response in MLCs. Because patients or animals with atherosclerosis were often associated with a low dose of blood circulation LPS [49,50], we used MLCs stimulated with LPS for further protocatechuic acid studies unless otherwise indicated to reflect the in vivo circumstances.

### 3.2. Protocatechuic Acid Reduces the Ratio of Nuclear to Cytoplasmic p-p65 Without Affecting Its Expression in MLCs

**Figure 3:**
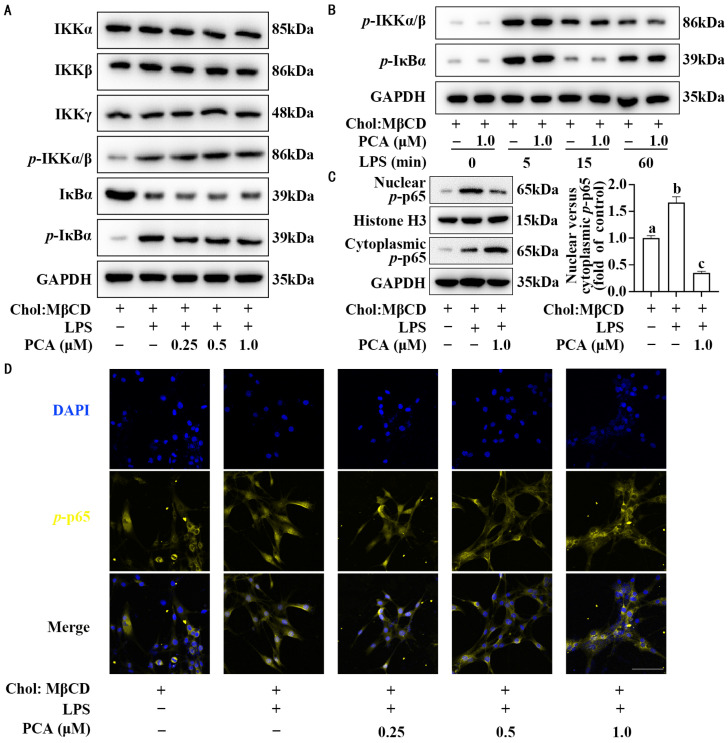
Protocatechuic acid reduces the ratio of nuclear to cytoplasmic *p*-p65 without affecting its expression in MLCs. (**A**) Representative Western blot of IKKα, IKKβ, IKKγ, *p*-IKKα/β, IκBα, and *p*-IκBα in MLCs incubated with protocatechuic acid for 72 h with LPS stimulation. (**B**) Western blot analysis was performed to evaluate the temporal expression dynamics of phosphorylated IKKα/β and IκBα in MLCs stimulated with different durations of LPS stimulation. Cells not treated with protocatechuic acid served as the control. (**C**) Representative Western blot and densitometric quantification of *p*-p65 translocation and distribution. (**D**) Representative immunofluorescent images depicting *p*-p65 localization (yellow). DAPI was used for nuclear counterstaining (blue). Scale bars: 100 μm. Values are shown as mean ± SEM. *n* = 6. Meanings associated with labels lacking shared letters exhibited significant divergence (*p* < 0.05).

As shown in Figure 3A, dose-response assays showed that protocatechuic acid exerted no significant effect on the total protein levels of IKKα/β/γ and IκBα and its phosphorylated form in MLCs stimulated with LPS. Time-response studies further showed that protocatechuic acid treatment failed to alter the abundance of phosphorylated IKKα/β and phosphorylated IκBα (Figure 3B). Interestingly, consistent Western blot and immunocytochemistry results revealed that protocatechuic acid changed the cellular distribution pattern of *p*-p65 in spite of no changes in total *p*-p65 abundance, in which the ratio of nuclear to cytoplasmic *p*-p65 was decreased (Figure 1C and Figure 3C,D).

### 3.3. Protocatechuic Acid Does Not Affect Importin Abundance and Cellular Distribution in MLCs

**Figure 4:**
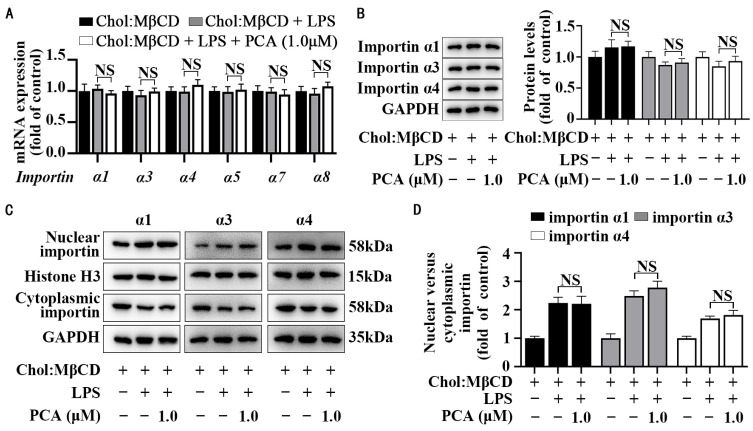
Protocatechuic acid does not affect importin abundance and distribution in MLCs. (**A**) Expression of the importin family was analyzed by qRT-PCR in MLCs incubated with protocatechuic acid for 72 h with LPS stimulation. (**B**) Representative Western blot and densitometric quantification of cellular importin. (**C**) Representative Western blot of importin abundance and (**D**) densitometric quantification of nuclear importin and cytoplasmic importin normalized to Histone H3 and GAPDH, respectively. Values are shown as mean ± SEM. *n* = 6. NS, nonsignificant (*p* ≥ 0.05).

The importin family plays an indispensable role in ferrying *p*-p65 from the cytoplasm to the nucleus. As depicted in Figure 4A,B, protocatechuic acid exerted no significant effect on either the transcriptional (mRNA) or translational (protein) expression levels of importin α1, α3, α4, α5, α7, or α8 in MLCs stimulated with LPS. Moreover, cellular location assays corroborated these findings, demonstrating that protocatechuic acid did not impact the ratio of nuclear to cytoplasmic importin α1, α3, or α4 (Figure 4C,D).

### 3.4. Protocatechuic Acid Regulates Cellular Distribution of Exportin-1 Without Affecting Its Expression in MLCs

**Figure 5:**
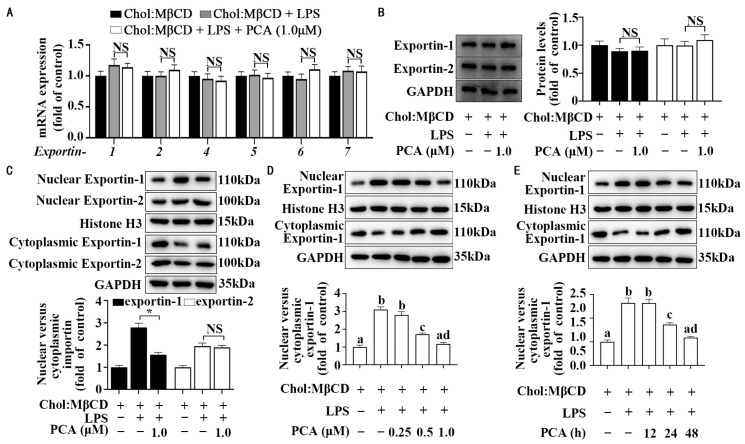
Protocatechuic acid regulates cellular distribution of exportin-1 without affecting its expression in MLCs. (**A**) Expression of the exportin family was analyzed by qRT-PCR in MLCs incubated with protocatechuic acid for 72 h with LPS stimulation. (**B**) Representative Western blot and densitometric quantification of cellular exportin. (**C**) Representative Western blot of exportin-1 and exportin-2 abundance and densitometric quantification of nuclear exportin and cytoplasmic exportin normalized to Histone H3 and GAPDH, respectively. (**D**) The dose-effect and (**E**) time-course experiments of protocatechuic acid on the nuclear-cytoplasmic distribution of exportin-1. Values are shown as mean ± SEM. *n* = 6. * Statistically significant difference compared to the control. Meanings associated with labels lacking shared letters exhibited significant divergence (*p* < 0.05). NS, nonsignificant (*p* ≥ 0.05).

Having shown no appreciable effect on the importin family, we next evaluated whether protocatechuic acid increased exportin activity, thus leading to a reduction in the ratio of nuclear to cytoplasmic *p*-p65 (Figure 3C). As depicted in Figure 5A,B, protocatechuic acid did not affect the mRNA and protein expression of exportin-1, 2, 4, 5, 6, or 7 in MLCs stimulated with LPS. Instead, protocatechuic acid significantly reduced the ratio of nuclear to cytoplasmic exportin-1 as compared with the control treatment; however, this compound did not affect the ratio of nuclear to cytoplasmic exportin-2 (Figure 5C). Time- and dose-response experiments further confirmed that protocatechuic acid could reduce the nucleocytoplasmic distribution of exportin-1 in MLCs stimulated with LPS (Figure 5D,E).

### 3.5. Exportin-1 Mediates the Anti-Inflammatory Property of Protocatechuic Acid in MLCs

**Figure 6:**
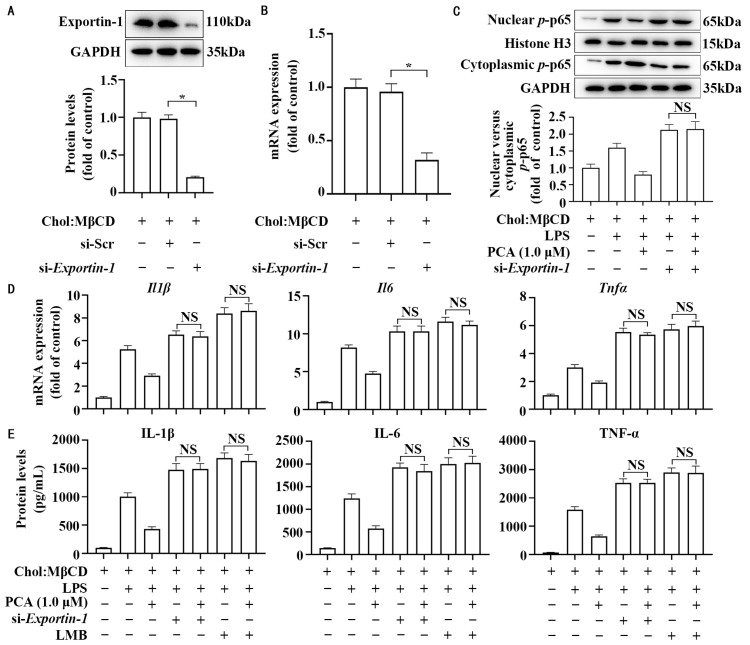
Exportin-1 mediates the anti-inflammatory property of protocatechuic acid in MLCs. (**A**,**B**) Knockdown efficiency of exportin-1 by siRNA strategies by Western blot (**A**) and qRT-PCR assays (**B**). (**C**) Representative Western blot and densitometric quantification of cellular *p*-p65 translocation and distribution. (**D**) mRNA expression of *Il1b*, *Tnfα*, and *Il6* by qRT-PCR. (**E**) Protein expression of IL-1β, TNF-α, and IL-6 by ELISA. Values are shown as mean ± SEM. *n* = 6. * Statistically significant difference compared to the control (*p* < 0.05). NS, nonsignificant (*p* ≥ 0.05).

To determine the function of exportin-1 in the anti-inflammatory property of protocatechuic acid, we employed small siRNA techniques and LMB (an inhibitor of exportin-1) to knock-down and inhibit exportin-1, respectively. Figure 6A,B showed that a reduction of approximately 70% in Exportin-1 expression was observed in cells transfected with *Exportin-1* siRNA compared to cells transfected with scrambled siRNA. Of note, *si-Exportin-1* abolished the anti-inflammatory effects of protocatechuic acid on the mRNA and protein expression of IL-1β, IL-6, and TNF-α (Figure 6D,E), as well as the ratio of nuclear to cytoplasmic *p*-p65 (Figure 6C). Similar to *si-Exportin-1*, LMB also abolished the protocatechuic acid effect (Figure 6D,E).

### 3.6. Dietary Protocatechuic Acid Reduces Inflammation Burden in MLCs Within Atherosclerotic Plaques and Inhibits the Transdifferentiation Progress

**Figure 7:**
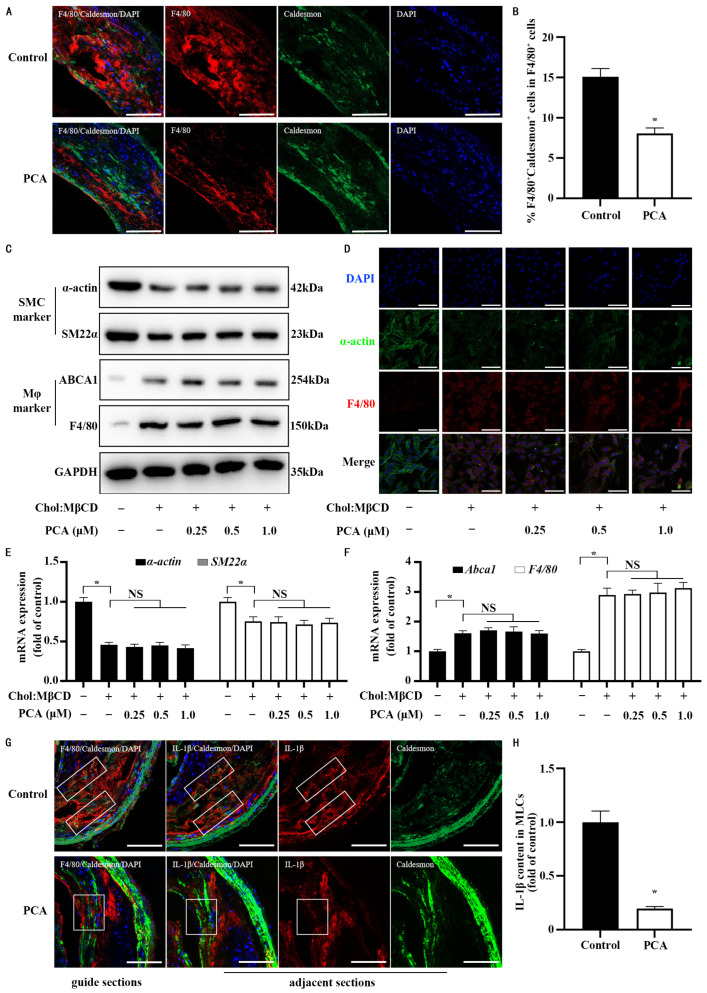
Dietary protocatechuic acid inhibits transdifferentiation of VSMCs into MLCs and also reduces inflammation burden in MLCs within atherosclerotic plaques. (**A**) Representative images of immunofluorescent staining of the brachiocephalic artery for F4/80 (red) and Caldesmon (green). DAPI was used for nuclear counterstaining (blue). (**B**) Quantitative analysis of the proportion of MLCs in macrophages within the brachiocephalic artery. *n* = 15. (**C**) Representative Western blot of VSMCs markers and macrophages markers expression in cells before and after cholesterol loading for 72 h. (**D**) Representative immunofluorescent staining images of F4/80 (red) and α-actin (green). DAPI was used for nuclear counterstaining (blue). (**E**) The mRNA expression of VSMC markers mentioned above. (**F**) The mRNA expression of macrophage markers mentioned above. *n* = 6. (**G**) Representative images of immunofluorescent staining of the brachiocephalic artery for F4/80 (red) or IL-1β (red) and Caldesmon (green). DAPI was used for nuclear counterstaining (blue). (**H**) Quantification of expression of IL-1β in MLCs (frame elected area) from fifteen fields of view per group. *n* = 15. Values are shown as mean ± SEM. Scale bars: 100 μm. * Statistically significant difference compared to the control (*p* < 0.05). NS, nonsignificant (*p* ≥ 0.05).

In order to test whether protocatechuic acid could recapture the in vitro findings on inflammation response in MLCs in vivo circumstances, 30-week-old ApoE^−/−^ were subjected to a 20-week dietary intervention consisting of an AIN-93G diet supplemented with or without protocatechuic acid. Using a dual-color colocalization strategy, double immunohistochemical staining with F4/80 (a macrophage marker) and Caldesmon (another VSMCs marker that is independent of cholesterol loading) was performed, and cells that co-expressed both markers were identified as MLCs (F4/80^+^Caldesmon^+^). Of note, protocatechuic acid feeding may inhibit the transdifferentiation of VSMCs into MLCs within atherosclerotic plaques, as evidenced by a reduction in the ratio of MLCs (F4/80^+^Caldesmon^+^) to whole macrophage populations (F4/80^+^) (47%, 95% confidence interval: 30.00 to 63.16) (Figure 7A,B). However, our in vitro findings showed that protocatechuic acid at any tested concentration did not affect the process of cell transdifferentiation of VSMCs into MLCs, which was evidenced by a non-significant difference in the abundance of VSMC markers (α-actin and smooth muscle 22α) and macrophage markers (F4/80 and ATP-binding cassette transporter A1) at the mRNA and protein levels (Figure 7C–F). Because our current study focused on the inflammation response in MLCs, we did not further dissect the effect of protocatechuic acid on the transdifferentiation of VSMCs into MLCs. The discrepancies between our in vivo and in vitro results are discussed in the Section 4. Notably, in addition to suppressing the transdifferentiation of VSMCs into MLCs, protocatechuic acid feeding inhibited IL-1β expression by 80% (95% confidence interval: 58.71–10.19) in MLCs within atherosclerotic plaques compared with the control diet (Figure 7G,H).

## 4. Discussion

Despite the well-established efficacy of statins in lowering plasma LDL cholesterol levels, residual cardiovascular risk in statin-treated populations remains high [51,52,53]. It is known that in addition to high levels of LDL cholesterol, non-resolving inflammation also plays a pivotal role in atherosclerosis development, highlighting that anti-inflammation strategies are effective in reducing the risk or severity of atherosclerotic cardiovascular events. For example, selective neutralization of molecules linked to inflammation, including IL-1β, significantly reduces recurrent cardiovascular events in post-myocardial infarction patients without modulating serum LDL cholesterol levels [54,55]. However, long-term inhibition of inflammation is notoriously known to increase the risk of opportunistic infections or cancer. As such, dietary polyphenols as a complementary and alternative medicine with a high safety have received great attention. Indeed, together with other groups work, our series of preclinical studies have shown that protocatechuic acid is effective in inhibiting atherosclerosis; however, the atheroprotective mechanism underlying this action remains largely unknown [24,35,36,37,38,56]. A lack of understanding of the atheroprotective mechanisms of dietary protocatechuic acid hampers the clinical application in patients with atherosclerosis.

In the current study, using MLCs that were established by the transdifferentiation of VSMCs, we observed that protocatechuic acid suppresses NF-κB activity and concordantly inhibits the expression of pro-inflammatory cytokines (IL-1β, IL-6, and TNF-α) in inflammatory MLCs by facilitating exportin-1-mediated transport of nuclear NF-κB into the cytoplasm. More importantly, dietary supplementation with protocatechuic acid for 20 weeks could also reduce inflammatory burden in MLCs within atherosclerotic plaques in ApoE^−/−^ mice. In addition, dietary protocatechuic acid could inhibit transdifferentiation of VSMCs into MLCs within plaques. These data thus may uncover a novel atheroprotective mechanism for protocatechuic acid.

While numerous studies have demonstrated that dietary polyphenols, including protocatechuic acid at 10–200 μM, could reduce inflammation burden in monocyte-derived macrophages by inhibiting IKK activation and/or IκBα degradation [40,57], two critical questions persist. On one hand, the dosage of polyphenols (10–200 μM) is far higher than that achieved in animals and humans after dietary polyphenol consumption or supplementation because of its low bioavailability [32,58]. On the other hand, the atheroprotective effects of polyphenols were exclusively evaluated in myeloid-derived macrophages, neglecting MLCs, an important subset of macrophages in the whole macrophage populations within murine and human atherosclerotic plaques [7,8]. In the current study, we demonstrated that protocatechuic acid at a dietary achievable dose (0.5–1 μM) could dose-dependently inhibit NF-κB activation and concordantly reduce inflammatory burden in MLCs. Interestingly, this effect was achieved independently of IKK activation and IκBα degradation, instead facilitating exportin-1-mediated transport of nuclear NF-κB into the cytoplasm, different from the effects of protocatechuic acid at supra-physiological dosage in myeloid-derived macrophages [40]. More importantly, we observed that long-term consumption of protocatechuic acid reduces inflammatory burden in MLCs within atherosclerotic plaques in ApoE^−/−^ mice. These findings thus broaden the atheroprotective mechanisms of dietary protocatechuic acid.

Of note, protocatechuic acid at physiologically achievable doses (0.5–1 μM) has been shown to inhibit the inflammation response in monocyte-derived macrophages by targeting MER proto-oncogene tyrosine kinase and mitogen-activated protein kinase 3/1, different mechanisms observed in MLCs [35,57]. This discrepancy highlights the concept that it is necessary to evaluate the anti-inflammatory mechanisms of protocatechuic acid in both monocyte-derived macrophages and MLCs. Because other polyphenols also could inhibit inflammation in the whole macrophage populations within atherosclerotic plaques, we call for a revisit to the anti-inflammatory effects and mechanisms of polyphenols at a dietary achievable dose in both monocyte-derived macrophages and MLCs. These studies would undoubtedly uncover the atheroprotective mechanisms of dietary polyphenols and, in turn, accelerate the process of translating laboratory discovery into patient care.

It is now generally accepted that not all macrophages in atherosclerotic plaques are of myeloid origin, as indicated by immunostaining for cell-type-specific markers or many other lineage tracing techniques [9,43]. Indeed, several independent groups have demonstrated that either cholesterol, 1-palmitoyl 2-(5-oxovaleroyl) phosphatidylcholine (one common oxidized phospholipid in oxidized LDL), or advanced glycation end products could promote transdifferentiation of VSMCs to MLCs [59,60,61,62]. Herein, using immunostaining for VSMC- and macrophage-related markers (Caldesmon and F4/80), we observed that dietary supplementation with protocatechuic acid inhibits transdifferentiation of VSMCs to MLCs within atherosclerotic plaques. However, our in vitro studies did not recapture this phenomenon, as protocatechuic acid did not affect cholesterol-loading-induced transdifferentiation of VSMCs to MLCs. This discrepancy may stem from the inherent limitations of in vitro systems in replicating in vivo aspects. Firstly, since that protocatechuic acid demonstrated inhibitory effects on macrophage-mediated oxidation of LDL and also reduced advanced glycation end products (both substances present in the atherosclerosis plaque) in type 2 diabetic rats [61,63], it is very likely that protocatechuic acid inhibits MLC formation by targeting these two stimuli rather than cholesterol in atherosclerotic plaques. Secondly, protocatechuic acid undergoes complex metabolism after oral absorption. It is thus possible that its metabolites play a role in inhibiting transdifferentiation of VSMCs into MLCs in vivo. Thirdly, there is a different duration between the in vitro (3 days) and in vivo (20 weeks) experiments, which may be responsible for the discrepancy.

As far as we are aware, there are no epidemiological and clinical trials evaluating the relationship between the intake of protocatechuic acid and atherosclerotic cardiovascular diseases. However, anthocyanins and proanthocyanidins that could be metabolized into protocatechuic acid after oral intake in humans have been shown to reduce the risk or severity of atherosclerosis [21,22,64]. Indeed, we and other groups have demonstrated the atheroprotective effect of protocatechuic acid in different animal models with atherosclerosis [24,35,36,37,38,56]. These findings thus suggest that protocatechuic acid might be an effective candidate to combat atherosclerosis in humans. Of note, the atheroprotective effect of protocatechuic acid is likely achieved independent of modulation of LDL cholesterol levels [56]. This highlights a possibility that the combination of statins and protocatechuic acid has a synergistic effect on atherosclerosis prevention and treatment. Further clinical trials with protocatechuic acid in patients with atherosclerosis are worthy of studies.

There are two limitations of the current study. First, after being absorbed from the gastrointestinal tract, protocatechuic acid is known to undergo an extensive biotransformation and produce several metabolites, such as sulfated and glucuronidated forms of protocatechuic acid and hippuric acid [45,64,65,66,67]. Therefore, it remains unclear whether protocatechuic acid and/or its metabolites are responsible for the anti-inflammatory function of protocatechuic acid. Second, it is not clear that specific signaling pathways mediate the effect of protocatechuic acid on exportin-1-mediated nuclear export of NF-κB. It is known that RanGTP, one type of small GTPase, is required to facilitate exportin-1 function [68]. Small GTPases are susceptible to redox regulation, where oxidation of cysteine residues within their conserved motifs can alter nucleotide-binding affinity and effector interactions [69]. Using human gastric adenocarcinoma cells, Lin et al. reported that protocatechuic acid could increase the abundance and activity of RhoB, a small GTPase [70]. This observation leads us to hypothesize that protocatechuic acid may affect exportin-1-mediated nuclear export of NF-κB through redox-mediated modulation of RanGTP activity.

## 5. Conclusions

In conclusion, our work is the first systematic inquiry to evaluate the effect of protocatechuic acid on the inflammatory burden in MLCs. We observed that protocatechuic acid is able to reduce the inflammation burden in MLCs likely through facilitating exportin-1-mediated transport of nuclear NF-κB into the cytoplasm and also inhibiting MLC formation within atherosclerotic plaques. Given that protocatechuic acid has a remarkable ability to inhibit atherosclerosis in animals with atherosclerosis by multiple mechanisms [24,35,36,37,38,56], we call for its translation study from preclinical studies to clinical trials in patients with atherosclerosis.

## Figures and Tables

**Table 1:** List of primary secondary antibodies used in this study.

Antibody	Cat#	Source
GAPDH	K200057m	Solarbio, Beijing, China
Histone H3	ab1791	Abcam, Cambridge, UK
IKKα ^1^	AF6012	Affinity Biosciences, Cincinnati, OH
IKKβ	AF6009	Affinity Biosciences, Cincinnati, OH
IKKγ	AF6495	Affinity Biosciences, Cincinnati, OH
phospho-IKKα/β	2697S	Cell Signaling Technology, Danvers, MA, USA
IκBα ^2^	4814S	Cell Signaling Technology, Danvers, MA, USA
phospho-IκBα	AP0707	Abclonal, Woburn, MA, USA
Caldesmon	85702	NOVUS, Oakland, NE, USA
α-actin	A2547/ab5694	Sigma-Adrich, St. Louis, MO, USA/Abcam, Cambridge, UK
SM22α ^3^	PA5-29767	Invitrogen, Waltham, MA, USA
F4/80	Ab16911	Abcam, Cambridge, UK
ABCA1 ^4^	ab18180	Abcam, Cambridge, UK
NF-κB/phospho-p65 ^5^	11014	SAB, Johannesburg, South Africa
IL-1β ^6^	AF-401-NA	R&D, Minneapolis, MN, USA
Importin α1	10819-1-AP	Proteintech, Rosemont, IL, USA
Importin α3	12463-1-AP	Proteintech, Rosemont, IL, USA
Importin α4	67892-1-IG	Proteintech, Rosemont, IL, USA
Exportin-1	66763-1-IG	Proteintech, Rosemont, IL, USA
Exportin-2	67306-1-IG	Proteintech, Rosemont, IL, USA
Alexa Fluor^®^ 647 anti-rabbit lgG	A21244	Invitrogen, Waltham, MA, USA
Alexa Fluor^®^ 488 anti-mouse lgG	A10680	Invitrogen, Waltham, MA, USA
Alexa Fluor^®^ 546 anti-goat lgG	A11056	Invitrogen, Waltham, MA, USA

^1^ IKK, inhibitor of NF-κB kinase; ^2^ IκBα, NF-κB inhibitor α; ^3^ SM22α, smooth muscle 22α; ^4^ ABCA1, ATP-binding cassette transporter A1; ^5^ NF-κB, nuclear factor-κB; ^6^ IL-1β, interleukin 1 beta.

**Table 2:** Primer sequences for real-time PCR.

Genes	Forward (5′−3′)	Reverse (5′−3′)
*Il6* ^1^	GTCCTTCAGAGAGATACAGAAACT	AGCTTATCTGTTAGGAGACCATTG
*Il1b* ^2^	CCAGCTTCAAATCTCACAGCAG	CTTCTTTGGGTATTGCTTGGGATC
*Tnfα* ^3^	CCCTCACACTCAGATCATCTTCT	GCTACGACGTGGGCTACAG
*Importin α1*	AAACGTCAGCTCCTTTCCTGAT	GGGATAGCACCTCCATCCAC
*Importin α3*	TCGGGAACTTCTGCACAGAC	ACACCGCTTGTTCACAAACATT
*Importin α4*	CCAGTGATCGAAATCCACCAA	CGTTTGTTCAGACGTTCCAGAT
*Importin α5*	TGGAGTTCCTCAAACGAAAAGAA	TTTGTCAGGACCCAAGCTGAT
*Importin α7*	AAGAACAATGCCTTAAACCCTGA	AGCAGACTATCAAACATGGCAG
*Importin α8*	CTACCTCAAAGGCTCCCAAAG	CGGAGTTGTAGACTGGAAGCAA
*Exportin-1*	CTGCTTGATTTCAGCCAAAAACT	GTATTTCGTGTTCATGTTCTGCG
*Exportin-2*	ATGGAGTCCTTCGTACAGCG	TCATTTGCATGGGTACTGCAC
*Exportin-4*	CGGTAACTGCAAGCGAGTCTT	CTCAGGACTTGGTTGGCGA
*Exportin-5*	TGTGCGAGGAGCTAGTGAAAG	TCTGACGATGGCAATTTGTGTT
*Exportin-6*	ATAAGATGGAAATCCGTAGCTGC	GGCGTCCAATATCAACGATAACT
*Exportin-7*	CGTGTCTCGGACAAACAACC	GCTTGAGTCACGAAAGTTGCC
*α-actin*	GCTTCGCTGGTGATGATGCTC	AGTTGGTGATGATGCCGTGTTC
*SM22α* ^4^	CCAACAAGGGTCCATCCTACG	ATCTGGGCGGCCTACATCA
*F4/80*	CCTGGACGAATCCTGTGAAG	GGTGGGACCACAGAGAGTTG
*Abca1* ^5^	GCTTCGCTGGTGATGATGCTC	AGTTGGTGATGATGCCGTGTTC
*Gapdh* ^6^	AACGACCCCTTCATTGAC	TCCACGACATACTCAGCAC

^1^ *Il6*, interleukin 6; ^2^ *Il1β*, interleukin 1 beta; ^3^ *Tnfα*, tumor necrosis factor α; ^4^ *SM22α*, smooth muscle 22α; ^5^ *Abca1*, ATP-binding cassette transporter A1; ^6^ *Gapdh*, glyceraldehyde-3-phosphate dehydrogenase.

## Data Availability

The original contributions presented in the study are included in the article, further inquiries can be directed to the corresponding author.

## Supplementary Materials

The following supporting information can be downloaded at: https://www.mdpi.com/article/10.3390/nu17061090/s1, Table S1: The ARRIVE guidelines 2.0: author checklist.

## Abbreviations

The following abbreviations are used in this manuscript:

ABCA1	ATP-binding cassette transporter A1
Chol:MβCD	cholesterol and methyl-β-cyclodextrin complexes
DAPI	4′,6-diamidino-2-phenylindole
IL	interleukin
LDH	lactate dehydrogenase
LMB	leptomycin B
LPS	lipopolysaccharide
Mφ	macrophage
MLCs	macrophage-like cells
MTT	3-(4,5-dimethylthiazol-2-yl)-2,5-diphenyltetrazolium bromide
NF-κB	nuclear factor kappa B
NS	nonsignificant
ox-LDL	oxidized LDL
PCA	protocatechuic acid
POVPC	1-palmitoyl 2-(5-oxovaleroyl) phosphatidylcholine
*p*-IκBα	phosphorylated NF-κB inhibitor α
*p*-IKK	phosphorylated NF-κB kinase inhibitor
siRNA	small interfering RNA
*si-Exportin-1*	*Exportin-1* small interfering RNA
*si*-Scr	scrambled small interfering RNA
VSMCs	vascular smooth muscle cells
SM22α	smooth muscle 22α

## References

[1] Libby P. (2021). The changing landscape of atherosclerosis. Nature.

[2] Soehnlein O., Libby P. (2021). Targeting inflammation in atherosclerosis—From experimental insights to the clinic. Nat. Rev. Drug Discov..

[3] Pan H., Ho S.E., Xue C., Cui J., Johanson Q.S., Sachs N., Ross L.S., Li F., Solomon R.A., Connolly E.S. (2024). Atherosclerosis Is a Smooth Muscle Cell–Driven Tumor-like Disease. Circulation.

[4] Bennett M.R., Sinha S., Owens G.K. (2016). Vascular Smooth Muscle Cells in Atherosclerosis. Circ. Res..

[5] Harman J.L., Jorgensen H.F. (2019). The role of smooth muscle cells in plaque stability: Therapeutic targeting potential. Br. J. Pharmacol..

[6] Swiatlowska P., Tipping W., Marhuenda E., Severi P., Fomin V., Yang Z., Xiao Q., Graham D., Shanahan C., Iskratsch T. (2024). Hypertensive Pressure Mechanosensing Alone Triggers Lipid Droplet Accumulation and Transdifferentiation of Vascular Smooth Muscle Cells to Foam Cells. Adv. Sci..

[7] Miano J.M., Fisher E.A., Majesky M.W. (2021). Fate and State of Vascular Smooth Muscle Cells in Atherosclerosis. Circulation.

[8] Pan H., Xue C., Auerbach B.J., Fan J., Bashore A.C., Cui J., Yang D.Y., Trignano S.B., Liu W., Shi J. (2020). Single-Cell Genomics Reveals a Novel Cell State During Smooth Muscle Cell Phenotypic Switching and Potential Therapeutic Targets for Atherosclerosis in Mouse and Human. Circulation.

[9] Shankman L.S., Gomez D., Cherepanova O.A., Salmon M., Alencar G.F., Haskins R.M., Swiatlowska P., Newman A.A., Greene E.S., Straub A.C. (2015). KLF4-dependent phenotypic modulation of smooth muscle cells has a key role in atherosclerotic plaque pathogenesis. Nat. Med..

[10] Allahverdian S., Chehroudi A.C., McManus B.M., Abraham T., Francis G.A. (2014). Contribution of intimal smooth muscle cells to cholesterol accumulation and macrophage-like cells in human atherosclerosis. Circulation.

[11] Vengrenyuk Y., Nishi H., Long X., Ouimet M., Savji N., Martinez F.O., Cassella C.P., Moore K.J., Ramsey S.A., Miano J.M. (2015). Cholesterol loading reprograms the microRNA-143/145-myocardin axis to convert aortic smooth muscle cells to a dysfunctional macrophage-like phenotype. Arterioscler. Thromb. Vasc. Biol..

[12] Wang Y., Dubland J.A., Allahverdian S., Asonye E., Sahin B., Jaw J.E., Sin D.D., Seidman M.A., Leeper N.J., Francis G.A. (2019). Smooth Muscle Cells Contribute the Majority of Foam Cells in ApoE (Apolipoprotein E)-Deficient Mouse Atherosclerosis. Arterioscler. Thromb. Vasc. Biol..

[13] Dubland J.A., Allahverdian S., Besler K.J., Ortega C., Wang Y., Pryma C.S., Boukais K., Chan T., Seidman M.A., Francis G.A. (2021). Low LAL (Lysosomal Acid Lipase) Expression by Smooth Muscle Cells Relative to Macrophages as a Mechanism for Arterial Foam Cell Formation. Arterioscler. Thromb. Vasc. Biol..

[14] Higashimori M., Tatro J.B., Moore K.J., Mendelsohn M.E., Galper J.B., Beasley D. (2011). Role of toll-like receptor 4 in intimal foam cell accumulation in apolipoprotein E-deficient mice. Arterioscler. Thromb. Vasc. Biol..

[15] Sun B., Dwivedi N., Bechtel T.J., Paulsen J.L., Muth A., Bawadekar M., Li G., Thompson P.R., Shelef M.A., Schiffer C.A. (2017). Citrullination of NF-kappaB p65 promotes its nuclear localization and TLR-induced expression of IL-1beta and TNFalpha. Sci. Immunol..

[16] Fagerlund R., Kinnunen L., Kohler M., Julkunen I., Melen K. (2005). NF-kappaB is transported into the nucleus by importin alpha3 and importin alpha4. J. Biol. Chem..

[17] Lin H.C., Li J., Cheng D.D., Zhang X., Yu T., Zhao F.Y., Geng Q., Zhu M.X., Kong H.W., Li H. (2021). Nuclear export protein CSE1L interacts with P65 and promotes NSCLC growth via NF-kappaB/MAPK pathway. Mol. Ther. Oncolytics.

[18] Azmi A.S., Uddin M.H., Mohammad R.M. (2021). The nuclear export protein XPO1—From biology to targeted therapy. Nat. Rev. Clin. Oncol..

[19] Boughanem H., Torres-Pena J.D., Arenas-de Larriva A.P., Romero-Cabrera J.L., Gomez-Luna P., Martin-Piedra L., Rodriguez-Cantalejo F., Tinahones F.J., Yubero Serrano E.M., Soehnlein O. (2025). Mediterranean diet, neutrophil count, and carotid intima-media thickness in secondary prevention: The CORDIOPREV study. Eur. Heart J..

[20] Nordmann A.J., Suter-Zimmermann K., Bucher H.C., Shai I., Tuttle K.R., Estruch R., Briel M. (2011). Meta-analysis comparing Mediterranean to low-fat diets for modification of cardiovascular risk factors. Am. J. Med..

[21] Yang Y., Ling W. (2025). Health Benefits and Future Research of Phytochemicals: A Literature Review. J. Nutr..

[22] Cassidy A., Mukamal K.J., Liu L., Franz M., Eliassen A.H., Rimm E.B. (2013). High anthocyanin intake is associated with a reduced risk of myocardial infarction in young and middle-aged women. Circulation.

[23] Wang D., Zou T., Yang Y., Yan X., Ling W. (2011). Cyanidin-3-O-beta-glucoside with the aid of its metabolite protocatechuic acid, reduces monocyte infiltration in apolipoprotein E-deficient mice. Biochem. Pharmacol..

[24] Wang D., Xia M., Yan X., Li D., Wang L., Xu Y., Jin T., Ling W. (2012). Gut microbiota metabolism of anthocyanin promotes reverse cholesterol transport in mice via repressing miRNA-10b. Circ. Res..

[25] Luo J.Y., Cheng C.K., He L., Pu Y., Zhang Y., Lin X., Xu A., Lau C.W., Tian X.Y., Ma R.C.W. (2022). Endothelial UCP2 Is a Mechanosensitive Suppressor of Atherosclerosis. Circ. Res..

[26] Alotaibi B.S., Ijaz M., Buabeid M., Kharaba Z.J., Yaseen H.S., Murtaza G. (2021). Therapeutic Effects and Safe Uses of Plant-Derived Polyphenolic Compounds in Cardiovascular Diseases: A Review. Drug Des. Devel Ther..

[27] Satheesh Babu A.K., Srinivasan H., Anandh Babu P.V. (2024). Breaking bugs: Gut microbes metabolize dietary components and modulate vascular health. Crit. Rev. Food Sci. Nutr..

[28] Garcia C., Blesso C.N. (2021). Antioxidant properties of anthocyanins and their mechanism of action in atherosclerosis. Free Radic. Biol. Med..

[29] Jing J., Guo J., Dai R., Zhu C., Zhang Z. (2023). Targeting gut microbiota and immune crosstalk: Potential mechanisms of natural products in the treatment of atherosclerosis. Front. Pharmacol..

[30] Lin W., Wang W., Yang H., Wang D., Ling W. (2016). Influence of Intestinal Microbiota on the Catabolism of Flavonoids in Mice. J. Food Sci..

[31] Vitaglione P., Donnarumma G., Napolitano A., Galvano F., Gallo A., Scalfi L., Fogliano V. (2007). Protocatechuic acid is the major human metabolite of cyanidin-glucosides. J. Nutr..

[32] Song J., He Y., Luo C., Feng B., Ran F., Xu H., Ci Z., Xu R., Han L., Zhang D. (2020). New progress in the pharmacology of protocatechuic acid: A compound ingested in daily foods and herbs frequently and heavily. Pharmacol. Res..

[33] Shi N., Chen F., Zhang X., Clinton S.K., Tang X., Sun Z., Chen T. (2017). Suppression of Oxidative Stress and NFkappaB/MAPK Signaling by Lyophilized Black Raspberries for Esophageal Cancer Prevention in Rats. Nutrients.

[34] Peiffer D.S., Wang L.S., Zimmerman N.P., Ransom B.W., Carmella S.G., Kuo C.T., Chen J.H., Oshima K., Huang Y.W., Hecht S.S. (2016). Dietary Consumption of Black Raspberries or Their Anthocyanin Constituents Alters Innate Immune Cell Trafficking in Esophageal Cancer. Cancer Immunol. Res..

[35] Zheng J., Li Q., He L., Weng H., Su D., Liu X., Ling W., Wang D. (2020). Protocatechuic Acid Inhibits Vulnerable Atherosclerotic Lesion Progression in Older *Apoe*^−/−^ Mice. J. Nutr..

[36] Ding H., Liu J., Chen Z., Huang S., Yan C., Kwek E., He Z., Zhu H., Chen Z.Y. (2024). Protocatechuic acid alleviates TMAO-aggravated atherosclerosis via mitigating inflammation, regulating lipid metabolism, and reshaping gut microbiota. Food Funct..

[37] Wang D., Wei X., Yan X., Jin T., Ling W. (2010). Protocatechuic acid, a metabolite of anthocyanins, inhibits monocyte adhesion and reduces atherosclerosis in apolipoprotein E-deficient mice. J. Agric. Food Chem..

[38] Li Q., Liu X., Du Y., Zhang X., Xiang P., Chen G., Ling W., Wang D. (2023). Protocatechuic acid boosts continual efferocytosis in macrophages by derepressing KLF4 to transcriptionally activate MerTK. Sci. Signal.

[39] Amini A.M., Spencer J.P.E., Yaqoob P. (2018). Effects of pelargonidin-3-O-glucoside and its metabolites on lipopolysaccharide-stimulated cytokine production by THP-1 monocytes and macrophages. Cytokine.

[40] Liu Y., Wang X., Pang J., Zhang H., Luo J., Qian X., Chen Q., Ling W. (2019). Attenuation of Atherosclerosis by Protocatechuic Acid via Inhibition of M1 and Promotion of M2 Macrophage Polarization. J. Agric. Food Chem..

[41] Vari R., D’Archivio M., Filesi C., Carotenuto S., Scazzocchio B., Santangelo C., Giovannini C., Masella R. (2011). Protocatechuic acid induces antioxidant/detoxifying enzyme expression through JNK-mediated Nrf2 activation in murine macrophages. J. Nutr. Biochem..

[42] Luo X., Xiao Y., Song F., Yang Y., Xia M., Ling W. (2012). Increased plasma S-adenosyl-homocysteine levels induce the proliferation and migration of VSMCs through an oxidative stress-ERK1/2 pathway in apoE^−/−^ mice. Cardiovasc. Res..

[43] Rong J.X., Shapiro M., Trogan E., Fisher E.A. (2003). Transdifferentiation of mouse aortic smooth muscle cells to a macrophage-like state after cholesterol loading. Proc. Natl. Acad. Sci. USA.

[44] Archbold H.C., Jackson K.L., Arora A., Weskamp K., Tank E.M., Li X., Miguez R., Dayton R.D., Tamir S., Klein R.L. (2018). TDP43 nuclear export and neurodegeneration in models of amyotrophic lateral sclerosis and frontotemporal dementia. Sci. Rep..

[45] Zheng J., Xiong H., Li Q., He L., Weng H., Ling W., Wang D. (2019). Protocatechuic acid from chicory is bioavailable and undergoes partial glucuronidation and sulfation in healthy humans. Food Sci. Nutr..

[46] Amin H.P., Czank C., Raheem S., Zhang Q., Botting N.P., Cassidy A., Kay C.D. (2015). Anthocyanins and their physiologically relevant metabolites alter the expression of IL-6 and VCAM-1 in CD40L and oxidized LDL challenged vascular endothelial cells. Mol. Nutr. Food Res..

[47] Percie du Sert N., Ahluwalia A., Alam S., Avey M.T., Baker M., Browne W.J., Clark A., Cuthill I.C., Dirnagl U., Emerson M. (2020). Reporting animal research: Explanation and elaboration for the ARRIVE guidelines 2.0. PLoS Biol..

[48] Trogan E., Fisher E.A. (2005). Laser capture microdissection for analysis of macrophage gene expression from atherosclerotic lesions. Methods Mol. Biol..

[49] Maitra U., Deng H., Glaros T., Baker B., Capelluto D.G., Li Z., Li L. (2012). Molecular mechanisms responsible for the selective and low-grade induction of proinflammatory mediators in murine macrophages by lipopolysaccharide. J. Immunol..

[50] Cani P.D., Amar J., Iglesias M.A., Poggi M., Knauf C., Bastelica D., Neyrinck A.M., Fava F., Tuohy K.M., Chabo C. (2007). Metabolic endotoxemia initiates obesity and insulin resistance. Diabetes.

[51] Chapman M.J., Zamorano J.L., Parhofer K.G. (2022). Reducing residual cardiovascular risk in Europe: Therapeutic implications of European medicines agency approval of icosapent ethyl/eicosapentaenoic acid. Pharmacol. Ther..

[52] Sampson U.K., Fazio S., Linton M.F. (2012). Residual cardiovascular risk despite optimal LDL cholesterol reduction with statins: The evidence, etiology, and therapeutic challenges. Curr. Atheroscler. Rep..

[53] Wong N.D., Zhao Y., Quek R.G.W., Blumenthal R.S., Budoff M.J., Cushman M., Garg P., Sandfort V., Tsai M., Lopez J.A.G. (2017). Residual atherosclerotic cardiovascular disease risk in statin-treated adults: The Multi-Ethnic Study of Atherosclerosis. J. Clin. Lipidol..

[54] Ridker P.M., MacFadyen J.G., Everett B.M., Libby P., Thuren T., Glynn R.J., Group C.T. (2018). Relationship of C-reactive protein reduction to cardiovascular event reduction following treatment with canakinumab: A secondary analysis from the CANTOS randomised controlled trial. Lancet.

[55] Ridker P.M., Everett B.M., Thuren T., MacFadyen J.G., Chang W.H., Ballantyne C., Fonseca F., Nicolau J., Koenig W., Anker S.D. (2017). Antiinflammatory Therapy with Canakinumab for Atherosclerotic Disease. N. Engl. J. Med..

[56] Hong K., Wang J., Kang X., Xue H., Gao Y., Liang H., Huang W., Zhan J., You Y. (2025). Ferulic acid and protocatechuic acid alleviate atherosclerosis by promoting UCP1 expression to inhibit the NLRP3-IL-1beta signaling pathway. Food Funct..

[57] Dejani N.N., Elshabrawy H.A., Bezerra Filho C., de Sousa D.P. (2021). Anticoronavirus and Immunomodulatory Phenolic Compounds: Opportunities and Pharmacotherapeutic Perspectives. Biomolecules.

[58] Stromsnes K., Lagzdina R., Olaso-Gonzalez G., Gimeno-Mallench L., Gambini J. (2021). Pharmacological Properties of Polyphenols: Bioavailability, Mechanisms of Action, and Biological Effects in In Vitro Studies, Animal Models, and Humans. Biomedicines.

[59] Han Z., Hu H., Yin M., Lin Y., Yan Y., Han P., Liu B., Jing B. (2023). HOXA1 participates in VSMC-to-macrophage-like cell transformation via regulation of NF-kappaB p65 and KLF4: A potential mechanism of atherosclerosis pathogenesis. Mol. Med..

[60] Chang M.K., Bergmark C., Laurila A., Horkko S., Han K.H., Friedman P., Dennis E.A., Witztum J.L. (1999). Monoclonal antibodies against oxidized low-density lipoprotein bind to apoptotic cells and inhibit their phagocytosis by elicited macrophages: Evidence that oxidation-specific epitopes mediate macrophage recognition. Proc. Natl. Acad. Sci. USA.

[61] Bao Z., Li L., Geng Y., Yan J., Dai Z., Shao C., Sun Z., Jing L., Pang Q., Zhang L. (2020). Advanced Glycation End Products Induce Vascular Smooth Muscle Cell-Derived Foam Cell Formation and Transdifferentiate to a Macrophage-Like State. Mediat. Inflamm..

[62] Pidkovka N.A., Cherepanova O.A., Yoshida T., Alexander M.R., Deaton R.A., Thomas J.A., Leitinger N., Owens G.K. (2007). Oxidized phospholipids induce phenotypic switching of vascular smooth muscle cells in vivo and in vitro. Circ. Res..

[63] Masella R., Vari R., D’Archivio M., Di Benedetto R., Matarrese P., Malorni W., Scazzocchio B., Giovannini C. (2004). Extra virgin olive oil biophenols inhibit cell-mediated oxidation of LDL by increasing the mRNA transcription of glutathione-related enzymes. J. Nutr..

[64] Gao Y., Tian R., Liu H., Xue H., Zhang R., Han S., Ji L., Huang W., Zhan J., You Y. (2022). Research progress on intervention effect and mechanism of protocatechuic acid on nonalcoholic fatty liver disease. Crit. Rev. Food Sci. Nutr..

[65] Chen W., Wang D., Wang L.S., Bei D., Wang J., See W.A., Mallery S.R., Stoner G.D., Liu Z. (2012). Pharmacokinetics of protocatechuic acid in mouse and its quantification in human plasma using LC-tandem mass spectrometry. J. Chromatogr. B Anal. Technol. Biomed. Life Sci..

[66] Liang S., Zhao Z., Liu L., Zhang Y., Liu X. (2024). Research Progress on the Mechanisms of Protocatechuic Acid in the Treatment of Cognitive Impairment. Molecules.

[67] Krzysztoforska K., Mirowska-Guzel D., Widy-Tyszkiewicz E. (2019). Pharmacological effects of protocatechuic acid and its therapeutic potential in neurodegenerative diseases: Review on the basis of in vitro and in vivo studies in rodents and humans. Nutr. Neurosci..

[68] Napolitano G., Esposito A., Choi H., Matarese M., Benedetti V., Di Malta C., Monfregola J., Medina D.L., Lippincott-Schwartz J., Ballabio A. (2018). mTOR-dependent phosphorylation controls TFEB nuclear export. Nat. Commun..

[69] Mitchell L., Hobbs G.A., Aghajanian A., Campbell S.L. (2013). Redox regulation of Ras and Rho GTPases: Mechanism and function. Antioxid. Redox Signal.

[70] Lin H.H., Chen J.H., Chou F.P., Wang C.J. (2011). Protocatechuic acid inhibits cancer cell metastasis involving the down-regulation of Ras/Akt/NF-kappaB pathway and MMP-2 production by targeting RhoB activation. Br. J. Pharmacol..

